# Novel Data on the Ecology of *Cochranella mache* (Anura: Centrolenidae) and the Importance of Protected Areas for This Critically Endangered Glassfrog in the Neotropics

**DOI:** 10.1371/journal.pone.0081837

**Published:** 2013-12-05

**Authors:** H. Mauricio Ortega-Andrade, Octavio Rojas-Soto, Christian Paucar

**Affiliations:** 1 Instituto de Ecología A.C., Red de Biología Evolutiva, Xalapa, México; 2 FundaciónEcoCiencia, Programa para la Conservación de Especies Amenazadas de Extinción en Ecuador, Quito, Ecuador; 3 Museo Ecuatoriano de Ciencias Naturales, Sección de Vertebrados, División de Herpetología, Quito, Ecuador; 4 Universidad Central del Ecuador, Escuela de Biología y Química, Quito, Ecuador; Texas Tech University, United States of America

## Abstract

We studied a population of the endangered glassfrog, *Cochranella mache*, at Bilsa Biological Station, northwestern Ecuador, from 2008 and 2009. We present information on annual abundance patterns, behavioral ecology, habitat use and a species distribution model performed with MaxEnt. We evaluate the importance of the National System of Protected Areas (SNAP) in Colombia and Ecuador, under scenarios of climate change and habitat loss. We predicted a restricted environmental suitability area from 48,509 Km^2^ to 65,147 Km^2^ along western Ecuador and adjacent Colombia; ∼8% of the potential distribution occurs within SNAP. We examined four aspects of *C. mache* ecology: (1) ecological data suggests a strong correlation between relative abundance and rainfall, with a high probability to observe frogs through rainy months (February–May); (2) habitat use and the species distribution model suggest that this canopy dweller is restricted to small streams and rivulets in primary and old secondary forest in evergreen lowland and piedmont forest of western Ecuador, with predictions of suitability areas in adjacent southern Colombia; (3) the SNAP of Colombia and Ecuador harbor a minimum portion of the predicted model of distribution (<10%); and (4) synergetic effects of habitat loss and climate change reduces in about 95% the suitability areas for this endangered frog along its distributional range in Protected Areas. The resulting model allows the recognition of areas to undertake conservation efforts and plan future field surveys, as well as forecasting regions with high probability of *C. mache* occurrence in western Ecuador and southern Colombia. Further research is required to assess population tendencies, habitat fragmentation and target survey zones to accelerate the discovery of unknown populations in unexplored areas with high probability of suitability. We recommend that *Cochranella mache* must be re-categorized as “Critically Endangered” species in national and global status, according with criteria and sub-criteria A4, B1ab(i,ii,iii,iv),E.

## Introduction

Amphibians are quickly declining worldwide and disappearing, especially in the tropical Andes [1]–[5]. This is true for Ecuador and Colombia, with ∼30% of species considered threatened by extinction, and over 18% considered as data deficient for categorization [2]. Changes in forest coverage have favored increment of habitat temperature, increase in the length of the rainy seasons, decrease of ground humidity and increase in the variability of inter-year rains in tropical forest of western Ecuador [6]. These variations can severely affect tropical amphibian communities [7]. Moreover, synergies on environmental factors and anthropogenic activities seem to have favored the presence of emerging diseases in amphibians, expansions of invader species and an accelerated process of deforestation, fragmentation and habitat loss in tropical forest [3], [5], [8]–[13]. Red lists of taxa and threatened categories proposed by IUCN [2], [14], [15] are mainly evaluated on the basis of quantitative and qualitative ―observed, inferred or suspected― demographic and ecological data, related to population sizes, distribution and occupancy of areas, regional threats and probabilities of extinction. However, it is necessary to assess those species with cryptic behavior, restricted distributions that are difficult to sample, limitations on the availability of localities records, and few data on the impact of habitat loss and relative population declines.

*Cochranella mache* Guayasamin & Bonaccorso, is an endemic species from northwestern Ecuador distributed on the tropical lowland forest and eastern slopes of Cordillera Mache–Chindul ( = Montañas de Mache-Chindul), a rather isolated mountain range in the northern portion of Cordillera de la Costa, and western slopes of the Cordillera de los Andes. Although the known range of *C. mache* is encompassed by protected areas, it is globally listed as Critically Endangered, because its extent of occurrence is considered to be less than 5000 km^2^ in five locations, where habitat undergoes constant extent declines, and its natural history remains poorly understood [16]. However, Ron *et al*. [17] considered this species nationally categorized as Data Deficient.

A wide range of methods has been used to evaluate the ecological niches and potential geographic distribution of species based on quantitative presence data, to infer species environmental requirements, predict distributions areas, and confront current and future biodiversity threats [13], [18]–[22]. Models generated with small sample sizes should be used to identifying, within a statistical and ecological context, regions that have similar environmental conditions which delimiting the potential distributional range for species. Thus, models based only in abiotic conditions ignoring other aspects of the evolutionary history of the species (biotic factors, vagility, local extinctions and the capacity of populations to adapt to new conditions) could result in an erroneous model for the geographic distribution of species [23], [24].

Despite the difficulty of obtaining detailed species’ inventories, biologists increasingly rely on distribution models to perform conservation strategies [22], [24]–[28]. However, there is a critical problem about threatened species in conservation biology: the need to know the essential environmental conditions which are occupied in a context of fundamental ecological niche theory [23], [29]–[31], based in poor information and a quite limited number of occurrence records [22].

To deal with these ecological aspects of this uncommon and endangered frog, the main goals of this study were: (1) to evaluate the influence of rainfall in the annual abundance pattern, (2) to provide new data on natural history and habitat use of *C. mache* at Bilsa Biological Station; and (3) to evaluate the importance for conservation of *C. mache* of National System of Protected Areas in Ecuador and adjacent Colombia, over a predicted model of distribution for the species in scenarios of climate change and habitat loss; all in order to provide solid current bases to assess its risk categorization and plan its further conservation.

## Methods

### Ethics statement

To conduct this study, we obtained the research permit N° 002 from the Governmental Authority (the Ministerio del Ambiente del Ecuador) approved by Ing. J. Trujillo, to work in localities along Esmeralda's province, northwestern Ecuador. The Bilsa Biological Station is a private reserve owned and managed by the Fundación Jatun Sacha; permits were granted in 2007 by the Executive Director, Dr. M. Asanza. The microhabitats where *C. mache* is known to inhabit were carefully surveyed at night time (2000 h–2300 h) by direct inspection. Manipulation of animals in the field was minimal in all cases. We measured each individual with a digital caliper (0.05 mm accuracy), recorded their vocalizations with an Olympus Digital voice Recorder VN-4100 and photographed them with a Samsung S85 digital camera. After we documented these ecological data, individuals were returned to the same site where they were found. During our study we did not make scientific collections in field, but additional specimens were found housed in the Museo Ecuatoriano de Ciencias Naturales (MECN, Quito, Ecuador), Fundación Herpetológica Gustavo Orcés (FHGO, Quito, Ecuador), and Museo de Zoología, (QCAZ, Quito, Ecuador).

### Study region and field surveys

Western Ecuador exhibits substantial environmental gradients, with diverse climates and complex topography due to the presence of the Mache–Chindul mountain range and its closeness to the Andes mountain range. These areas are mainly covered by Foothill evergreen forests and lowland evergreen forests, as part of the Western Ecuadorian Biogeographic Province [32]. In this region, the dry season occurs from July to December and the wet season from January to June, with an annual average rainfall of 1500–2000 mm [33]. In order to find the species in the field, we decided to monitor populations in Dos Bocas, the type locality of *C. mache*, and Piscinas, Rompe-frente and Río Duchas, three other rivulets at Bilsa Biological Station BBS (N°0.359170, W°79.700560; altitudinal range from 300–750 m a.s.l.), in Mache-Chindul Mountain range, northwestern Ecuador. These rivulets were previously pre-selected by direct inspection by H.M.O-A in 2006, based on ease of accession and local facilities to do field work. Three of them (Piscinas, Rompe-frente, Dos Bocas) are rivulets with primary and well-preserved forests, but Río Duchas is covered mainly by old secondary forest. The surrounding areas have been largely deforested for small-scale agriculture, although several fragments of primary and secondary forests remain in the Bilsa Biological Station [34]. These sites are located in a range of 1000 – 2000m from each other, therefore are considered statistically as nested samples in regard to avoid random effects or pseudoreplication.

Field surveys were made through five sampling sessions during 2008 (13–25April, 9–17 May, 11–18 October) and 2009 (20–29 January and 14–23 April). Each day, the microhabitats where *C. mache* is known to inhabit were carefully surveyed at night (2000 h–2300 h), in a randomly selected rivulet. Sampling effort per day was 6 person-hours (2 people X 3 hours) and the cumulative sampling effort for the study was 300 person-hours (6 person hours X 50 days of surveys). We registered data traits on each frog encountered as follows: snout-vent length (SVL, mm), sex (direct inspection of vocal sac, gravid females), reproduction behavior (mating and egg deposition), and activity. To assess the use of microhabitat, we recorded the substrates on which each specimen was observed, and we assigned them to one of the following types of vertical forest stratification: understory (<2 m above ground), midstory (2–10 m), and canopy (>10 m). In spite of our effort, the canopy strata may be under-sampled due by sampling limitations. To assess the relation between relative abundances (RA =  # frogs observed/3 hours) and daily rainfall (mm) through samplings, we performed a Non-parametric Correlation and generated a Maximum Likelihood Linear Regression Model using R software (http://www.r-project.org/). Differences among relative abundances of individuals per rivulet and vegetation type were tested with Generalized Linear Models (GLM), with Poisson distribution, and a *P*-value was calculated using R software. Differences on snout-vent length between males and females were compared with a non-parametric Mann-Whitney test (U’), whereas the preferences in vertical forests stratification were tested with a Chi-squared test (X^2^), developed in PAST 1.86b software [35].

The call described herein for *C. mache* was recorded using an Olympus Digital voice Recorder VN-4100. Distance between recorder and frog was ∼2 m. Five parameters were measured to describe the structure of each call, as follows [36], [37]: (1) Call length =  time from beginning to end of one call, measured from waveform in milliseconds; (2) dominant frequency =  frequency in call containing the greatest amount of energy determined from the entire call; (3) call rise time  =  time from beginning of the call to point of maximum amplitude; (4) interval between calls  =  time from ending of a call and beginning of next call; and (5) call rate  =  total number of calls-1/time from beginning of first call to beginning of last call, all relativized to 60 seconds. Measurement of fundamental frequency follows the technique used by Hutter and Guayasamin [38]. A note is considered herein as an element with similar acoustic structure, whereas a pulse is the repetition of the calling note along the repertory. The sonogram was produced and analyzed using the program Raven Pro 1.5. After a preliminary review of the original sound spectrogram, we have applied a filter band-pass between 4300 and 7000 Hz in order to reduce noise and maximize the measurement of acoustic parameters. The dominance frequency was calculated with a size of 1500 samples in the spectrogram window. Recordings are deposited in the Sound Archive of the Museo Ecuatoriano de Ciencias Naturales and Museo de Zoología of Pontificia Universidad Católica del Ecuador and will be available at Amphibia Web Ecuador (http://zoologia.puce.edu.ec/vertebrados/anfibios/).

### Species Distribution Model

Our database gathers data from the seven known localities reported by different authors [16], [34], [39], [40], and is shown in Table 1. Geographic coordinates are provided in decimal degrees, based on the WGS 84 datum. Each locality was carefully geo-positioned (lat–long coordinates) to correct the geographic coordinates of imprecise localities and to eliminate any inconsistencies or duplicates. The corroboration was based on the revision of databases and specimens housed in the Fundación Herpetológica Gustavo Orcés (FHGO); Museo Ecuatoriano de Ciencias Naturales (DHMECN); and Museo de Zoología, Pontificia Universidad Católica del Ecuador (QCAZ), Quito, Ecuador.

**Table 1:** Localities of occurrence for *Cochranella mache* in northwestern Ecuador.

Locality	Latitude	Longitude	m.a.s.l.
3 km NW from Quinindé	N°0.350000	W°79.483330	150
Alto Tambo, Carolina River	N°0.912748	W°78.618827	453
Bilsa Biogical Station	N°0.359170	W°79.700560	800
Canandé Biological Reserve	N°0.447720	W°79.148080	270
Comunidad San Salvador*	N°0.496710	W°79.852980	38
Hacienda Shangrilá*	N°0.186300	W°79.030190	499
Monte Saíno, San Francisco del Cabo	N°0.704560	W°80.025330	100

(*) New records reported in this article.

We modeled the habitat suitability for *C. mache*, with the MaxEnt Software version 3.3.3a. [20], [41], [42]. MaxEnt estimates the probability of distribution that has maximum entropy applying the following principle: the expected value for each feature (*i.e.* climatic variables) must equal the empirical average value for points relating to known presence. The algorithm performs a certain number of iterations until reaching a convergence limit. The final map represents a favorability rating ranging from 0 (unsuitable) to 1 (perfectly adequate) [30]. The program uses two input resources: localities of the species record (presence-only data) and digital layers of the environmental conditions of a given area. For a first explorative model, we used the localities dataset described above, in combination with 19 climate layers from the WorldClim project [43]. In a second modeling exercise, we generated the species distribution using only the environmental variables that were relevant according to the Jackniffe test, calculated by Maxent [44]. This allowed us to reduce over-fitting on the distribution models generated for this species [45]. Resolution grid cell size, or pixel size, was 0.0083 degrees, which correspond nominally ∼1 km^2^ in each raster.

To aid model validation and interpretation, it is usually desirable to distinguish ‘suitable’ from ‘unsuitable’ areas by setting a decision threshold above which model output is considered to be a prediction of the species presence. There is no set rule to set these thresholds because its selection depends on the data used or the objective of the map, and will vary from species to species [46]. Then, we followed the settings suggested by Pearson *et al.* [24] for small numbers of occurrence records, where multiple predictions (seven from our database) were made with one of the observed localities excluded in each case. For each prediction, two threshold decisions were applied (Minimum training presence and Fixed cumulative value of 20), and the ability to predict the excluded locality was tested. A *P*-value was then calculated for the overall model across the set of jackknife predictions using the script made by Pearson *et al.* [24].

The overall predictive model of distribution for the species was generated with all the localities from the database, 5000 iterations and specified to the program not to do clamping and not to extrapolate, in order to avoid artificial extrapolations on the extremes of the ecological variables. All other parameters were maintained as default settings in MaxEnt. The logistic format was used to obtain the values of habitat suitability (continuous probability from 0 to 1), and then converted to binary presence-absence values. The results for both threshold values (MTP and T20) were represented by two distinct colored areas in the geographic space, whereas MTP is a sub-conjunct in T20.

To improve, accurate and validate the predicted distribution model of the species, we based our ecological criteria in classifications for Vegetation Formations [33], Terrestrial EcoRegions [47], and Biogegraphic Provinces [32] in northwestern South America. These criteria were used in combination because adding historical, evolutive and ecological factors into the model generated for delimitate the species distribution area. To assess the effect of deforestation, we used a representation of vegetation land cover included in two proposals of classification system for continental Ecuadorian and Colombian ecosystems [48], [49]. In order to evaluate the effect of habitat loss, we only consider the category called “natural forests” from both classification systems, whereas “perturbed areas” include categories which represent urban areas, secondary forests, deforested areas, farming areas and pasture land for cattle raising. We also evaluate the importance of the National System of Protected Areas (SNAP, from Spanish) in Ecuador and Colombia comparing the species distribution area predicted by our *C. mache* model against the area currently included in the SNAP, using layers downloaded from ProtectedPlanet.net [50]. The presence extension range was created from a geometric convex hull polygon that resulted from the union of all points on verified localities. Using a polygon could underestimates the presence extension range of the species, especially since *C. mache* was recently described and additional localities of occurrence are expected to record. Anyway, we decide to apply this method because is one of the techniques commonly used to evaluate the extension range of occurrence for threatened species [51]. Spatial analyses and map algebra were developed with ArcMap 10 Software, whereas the convex hull polygon was calculated from Minimum Bounding Geometry routine in ArcTool Box [52].

### Species Distribution Model in Scenarios of Future Global Climate Change

Albeit the potential problems associated with the use of global climate change scenarios at local scales [53], we find then useful as they show possible tendencies in future potential distribution of species, in this case *Cochranella mache*. For this study, we used General Circulation Models (GCMs) developed by the Canadian Centre for Climate Modeling Analysis (CCCMA) consisting in a coupled atmosphere-ocean GCM. Therefore, two (CCCMA-A2 and CCCMA-B2) of the four climatic scenarios established by the International Panel for Climate Change [IPCC, 54], were used. GCMs results were downloaded from the WorldClim website as digital layers and are based on the same 19 bioclimatic variables (http://www.ipcc-data.org/sres/cgcm1_info.html) used to generate the species distribution model; all include atmospheric and oceanic components such as heat flux, wind stress, salinity, levels of greenhouse gasses, soil drainage and moisture, snow and ice temperature, snow depth, cloud water content, effective radius of cloud droplets, vegetation type, among others.

The climatic models used herein are in various ways the two more moderate scenarios, in a framework of social and environmental strategies based on local and regional organization, being more likely probable to occur in the future world, rather than international cooperation and social equity schemes. Both climatic scenarios were generated in recent years by the IPCC, considering the main forces that may determine future emissions, and describe dynamic changes occurring in different directions; from demographic to technological and economic aspects, as well as the existence of sinks and sources of greenhouse gasses, the development of alternative schemes for energy production and the occurrence of changes in land use. A detailed description of each model is available in the IPCC Special Report on Emission Scenarios [54]. To predict the persistence of *Cochranella mache*’s ecological niche in a relatively close future, we decided to use the 2050 temporal horizon, since the species faces multiple conservation problems that may seriously threat its survival in the short term, and also because of the higher levels of uncertainty associated to more distant scenarios. Thus, we generated two maps representing the potential distribution of *C. mache* in 2050, under the GCM’s Canadian CCCMa-A2 (“pessimistic”) and CCCMa-B2 (“optimistic”) climatic scenarios.

## Results

### Abundance

**Figure 1:**
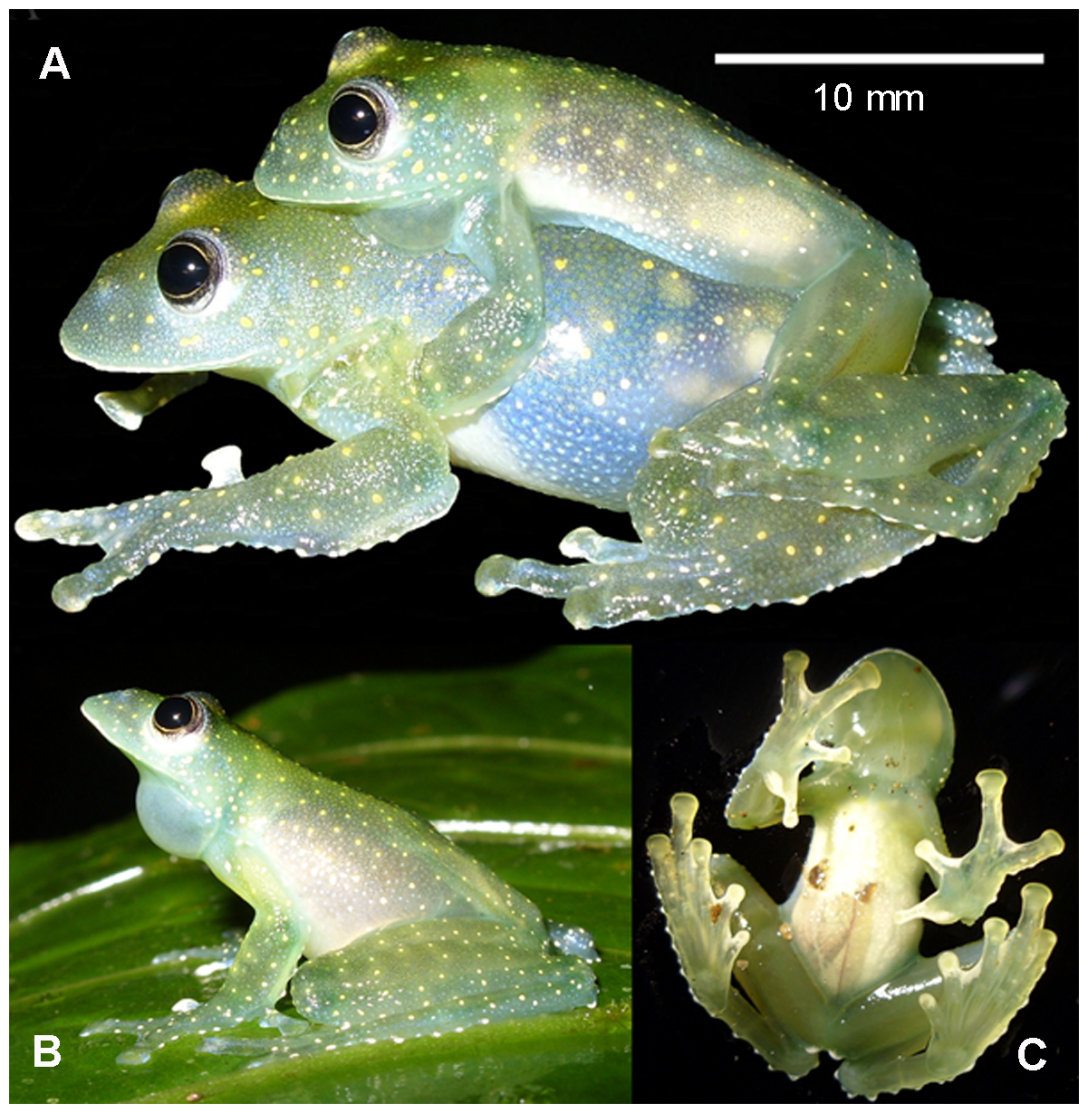
Cochranella mache. Amplectant pair captured on the April 22, 2009 Rompe-frente rivulet (A) and a calling male (SVL = 26.4 mm, not collected) showing its profile and ventral views, photographed on the April 20, 2009 Rompe-frente rivulet, Bilsa Biological Station, northwestern Ecuador (B, C). Photographs by Christian Paucar.

**Figure 2:**
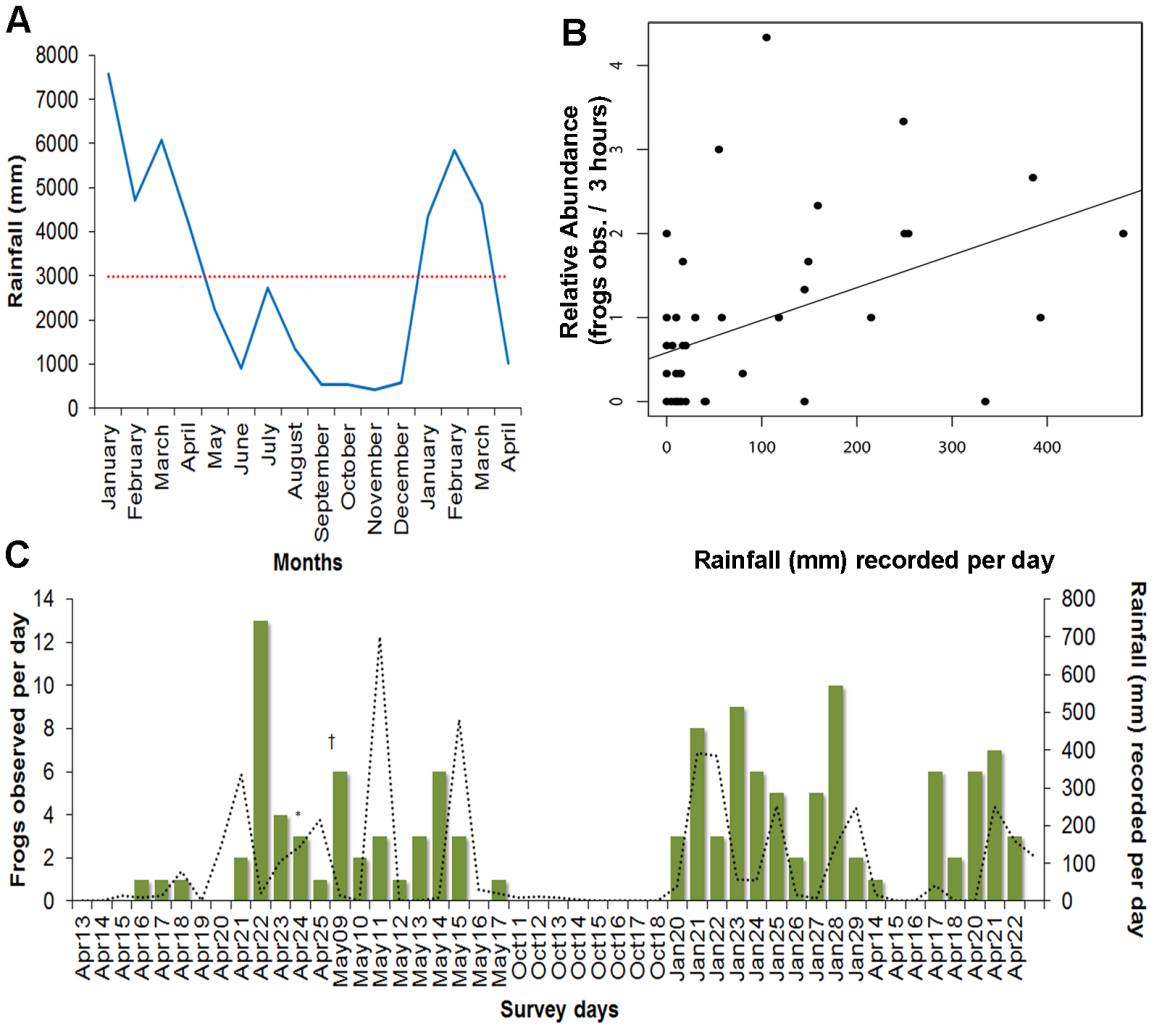
Rainfall, abundance and records of reproductive specimens of *Cochranella mache* in Bilsa. (A) Rainfall records through years 2008–2009 in Mache-Chindul Mountain range. (B) Correlation (*N* =  48, *r* = 0.456, *r^2^* = 0.21, *P*< 0.001) and Maximum Likelihood Linear Regression Model (Y =  0.5823+0.00387 (X), between relative abundance (Individuals observed/3 hours per day) of *C. mache* and rainfall (mm). (C) Number of observed individuals of *C. mache* and daily rainfall (mm) recorded through sampling days at Bilsa. The register of an amplectant pair (*) and a specimen recorded for vocalization analysis (†) are shown in (C).

We recorded 129 individuals of *C. mache* at Bilsa Biological Station throughout 2008 and 2009 surveys (Fig. 1). The overall average of observed frogs during the rainy season was 3.2 individuals per sampling day, and none was recorded in the dry season (October). We found an annual increase rainfall pattern from January to April (range: 4224–6080 mm), and a constant rainfall pattern that stays below the average annual rainfall from May to December (range: 422–2727 mm). We also found a significant correlation value (*N* =  48, *r* = 0.456; *P*< 0.001) between daily rainfall and relative abundance, with increase of individuals observed from January to April (Fig. 2). The maximum likelihood linear model fits by the equation (Fig. 1B): Relative abundance (Frogs observed/3 hours per day)  =  0.5823+0.00387(rainfall); 21% of the total variation in the response of relative abundance is explained due variation in daily rainfall. We did not find differences in the observational probability of specimens among vegetation types (*P = * 0.57) or rivulets (*P = * 0.97) in Dos Bocas (0.33 ind./person-hour), Duchas (0.53 ind./person-hour), Piscinas (0.57 ind./person-hour) and Rompe-frente (0.61 ind./person-hour).

### Natural history

**Figure 3:**
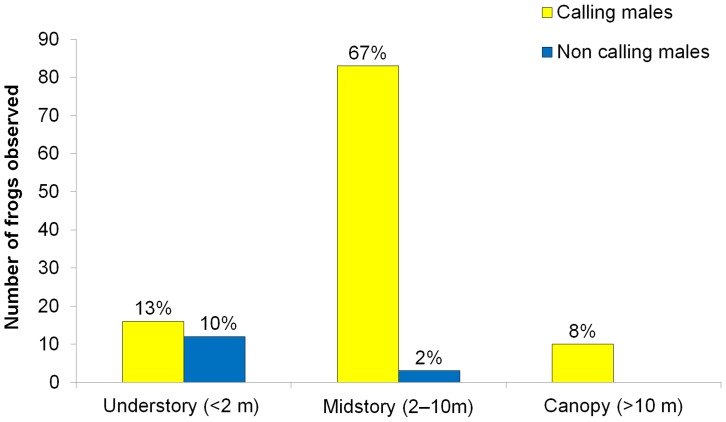
Vertical forest stratification use. Histogram of frequency (percentages) of males of *C. mache*, categorized by vertical forest stratification at Bilsa Biological Station, northwestern Ecuador.

*Cochranella mache* (Fig. 1) is a medium sized frog, females (*N* = 3; 28–33.4 mm; 31.4±2.96 mm) are significantly larger than males (23–27.0 mm; 25.28±1.02 mm; U’ = 0; *P*<0.001; *N* = 38). A gravid female (SVL 33.4 mm) with about 30 eggs was observed on a leaf, 1.85 m above ground, the night of 22 April 2009 in Rompe-frente rivulet. That same night, we found a pair in axillar amplexus perched on a leaf, 1.5 m above the ground (Fig. 1A). Throughout the rainy season, males were common to hear, calling from the upper side of leaves or branches on trees and bushes. Frequently, chorus of 4 or 5 calling males were heard from the forest vegetation, within a relatively small area, spaced ∼10-m apart, on overhanging vegetation along the rivulets (range: 0–6 m, average ± sd: 0.7±1 m). About 80% of individuals were observed in the upper part of the forest (Fig. 3), with a notable preference of the midstory over the understory and canopy (X^2^ = 76.3, *P*<0.001).

**Figure 4:**
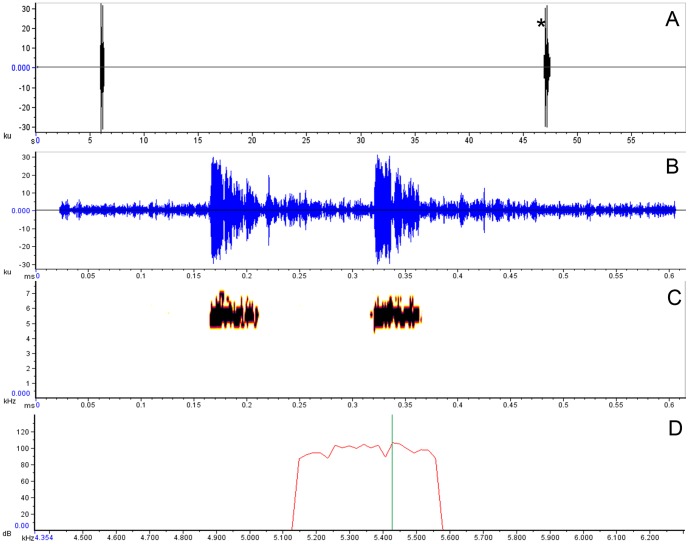
Predation in *Cochranella mache*. (A) The snake *Leptodeira septentrionalis* and (B) a Ctenid spider at Bilsa Biological Station, northwestern Ecuador. Photographs by C. Paucar.

We recorded two calls from a male at night (2254h) on May 9, 2008, in Rompe-frente rivulet (Fig. 1B). It was calling from the upper side of a dead *Heliconia* leaf, about 2.5 m above ground, horizontally separated from the rivulet’s water by a distance in about 3m. Each call consists of two pulses, repeated 0.107−0.130 second intervals, with duration of 0.038±0.008 (0.029−0.049) seconds, and the call rise time in 0.013±0.007 (0.002−0.021) seconds. The dominant frequency is 5410.2±17.9 (5383.3−5426.4) Hz, being part of the fundamental frequency (5139.5−6058.8 Hz); the harmonics are not visible in the audio-spectrogram. The rate is 1.46 calls per minute, repeated within ∼40.8 seconds intervals (Fig. 4).

**Figure 5:**
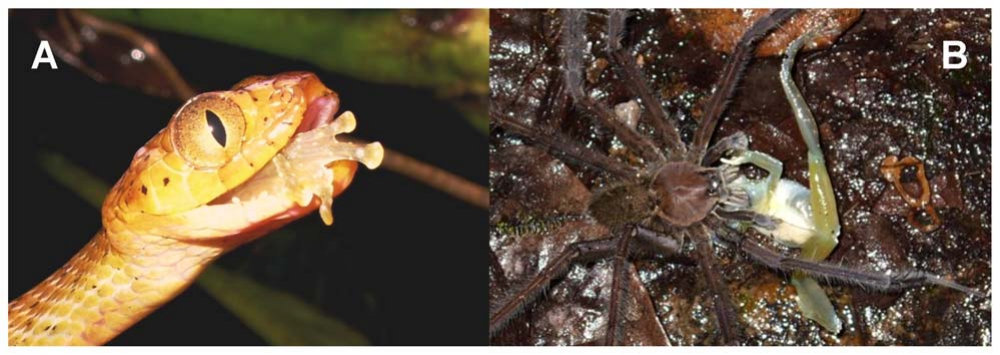
Call of *Cochranella mache.* (A) Section oscillogram, (B) call oscillogram, (C) spectrogram and (D) power spectrum generated from a calling male recorded at 2254 h, 9 May 2008, Rompe-frente rivulet at Bilsa Biological Station. The second call (*) was used to analyze acoustic data in (B−D). Green line in (D) marks the peak frequency (5426.4 Hz) in the band spectrum.

We also report predation behavior on *C. mache* from the colubrid snake *Leptodeira septentrionalis arcorum* Schmidt & Walker (Colubridae), observed at 2249h during the night on January 23, 2009, in Piscinas rivulet. The specimen (not sexed) was captured about 2.3 m above ground and eaten by the head (Fig. 5A). Besides, a female was observed captured by the neck by a *Ctenus* sp. spider (Aranae: Ctenidae), when the frog jumped to the water in Piscinas rivulet, on the night of January 23, 2009. The spider held the frog belly- up with legs outstretched (Fig. 5B). The frog, was struggling feebly. The spider had bitten the frog on the left gular region and shoulder, which showed a hemorrhage-like discoloration. After approximately 5 minutes, the frog ceased struggling, and the spider, still holding the frog by the shoulder, repositioned it. The frog tried to escape, striking his hind limbs toward the spider and turning over onto its back, with its arms. After 8–10 minutes, the frog was immobilized and eaten by the spider.

### Potential distribution model

**Figure 6:**
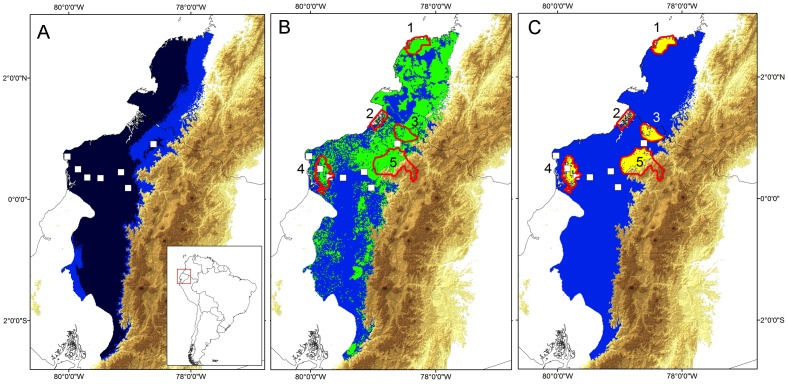
Potential distribution model of *Cochranella mache.* (A) Potential distribution model is shown with two thresholds, the minimum training presence approach (MTP, dark blue) and Fixed cumulative value of 20% (T20, light blue); (B) Remnant potential distribution model with natural forests (green areas); (C) Remnant potential distribution model predicted in the National System of Protected Areas of Ecuador and Colombia (yellow areas bordered by red). Squares symbols represents localities used to generate the models. Numbers in (B-C) correspond to Sanquianga Natural National Park (1), Manglares-Mataje Ecological Reserve (2), Awa Forest Reserve (3), Mache-Chindul Ecological Reserve (4), and Cotacachi-Cayapas Ecological Reserve (5). Note a critical reduction (∼69%, in green) of the best-predicted potential distribution model (T20 threshold), when it was filtered by areas with natural forests (B), and over 95% when it was filtered by Protected Areas in Ecuador and Colombia (C). The model was generated with the most important environmental variables in MaxEnt (See Materials and methods for details, but Table 2).

The effective presence extension range result in a convex hull polygon within ca. ∼6,940 km^2^, located in northwestern Ecuador. MaxEnt approach algorithm predicted an environmental suitability area for *C. mache* from 48,509 Km^2^ to 65,147 Km^2^ (depending on the MTP or T20 thresholds, respectively), with an elongated and curved shape, located from western Ecuador to the southern limit of adjacent Colombia (Fig. 6A). The most important environmental variables to model the suitability areas for *C. mache* are shown in Table 2. The projected potential distribution model of *C. mache* (Fig. 6B-C) was trained using seven localities, showing high and significant success rates in jackknife tests with a threshold of 20% (T20 = 0.12, *P = * 0.01), rather than the minimum training presence (MTP = 0.31, *P = *0.16). The minimum training presence approach (MTP) can be interpreted ecologically as identifying pixels predicted as being at least as suitable as those pixels where the species has been recorded, whereas the Fixed cumulative value (T20) rejected only the lowest 20% of possible predicted values in the model. The first approach in this case was more conservative and strict than the second, in which a larger predicted area is incorporated through the model. Then, the MTP model is a sub-conjunct in the geographic space of T20 model. The potential distribution model is related with Tropical evergreen lowland forest of Ecuador and Colombia (∼66%), Tropical piedmont forests in the western slopes of Andes (∼28%), Tropical evergreen low montane forest of western Andes (∼5%) and Tropical montane forest in the Coastal Range of Ecuador (1%).

**Table 2:** Summary of the most important environmental variables selected by MaxEnt, with relative contributions in the ecological niche model of *Cochranella mache.*

Abbreviation	Environmental Variable	Percent contribution
Bio 2	Mean diurnal temperature range (mean of monthly [maximum temperature – minimum temperature])	62.0
Bio 18	Precipitation of warmest quarter	19.9
Bio 19	Precipitation of coldest quarter	11.7
Bio 14	Precipitation of the driest month	4.6
Bio 6	Maximum temperature of warmest month	1.6
Bio 3	Isothermality (BIO2/BIO7 x 100)	0.1
Bio 7	Temperature range (maximum temperature of the warmest month – minimum temperature of the coldest month)	0.1

Predicted and remnant areas of the potential distribution model of *C. mache* in protected areas along western Ecuador and adjacent Colombia are detailed in Tables 3 and 4. The model resulted in isolated fragments of natural forest included in protected areas (∼5243 km^2^; Fig. 6) with a reduction which represents ∼92% of the potential distribution model (Table 3).

**Table 3:** Potential distribution area of Cochranella mache and percentage of such distribution under two scenarios of climate change (CCCMa-A2, CCCMa-B2), effect of habitat loss and remnant model included in protected areas in northwestern Ecuador and southern Colombia.

Model	T20 (∼km^2^)	%	MTP (∼km^2^)	%
Potential distribution area	65147	100	48509	100
Area of the model with natural forests	19942	31	11987	25
Area of the model included in Protected Areas	5243	8	3133	6
Remnant model within Protected Areas and natural forests	3096	5	1701	4
Model under CCCMA-A2	51533	79	37965	78
Area of the model with natural forests	14095	22	7793	16
Area of the model included in Protected Areas	3967	6	2411	5
Model under CCCMA-B2	56576	87	44082	91
Area of the model with natural forests	16281	25	10540	22
Area of the model included in Protected Areas	4224	6	3388	7

Logistic threshold decision was applied on models with a Minimum training presence (MTP) and Fixed cumulative value of 20% (T20, but see Materials and Methods).

**Table 4:** Potential distribution model of glassfrog *Cochranella mache*, in Km^2^ (percentages), predicted for protected areas in western Ecuador (Ecu) and southern Colombia (Col).

Protected area	T20	MTP
1. Sanquianga Natural National Park (Col)	904 (17%)	904 (29%)
2. Manglares-Mataje Ecological Reserve (Ecu)	205 (4%)	205 (7%)
3. Awa Forest Reserve (Ecu)	937 (18%)	106 (3%)
4. Mache-Chindul Ecological Reserve (Ecu)	1433 (27%)	1433 (46%)
5. Cotacachi-Cayapas Ecological Reserve (Ecu)	1764 (34%)	465 (15%)
Total predicted model in Protected Areas	5243 (100%)	3113 (100%)

**Figure 7:**
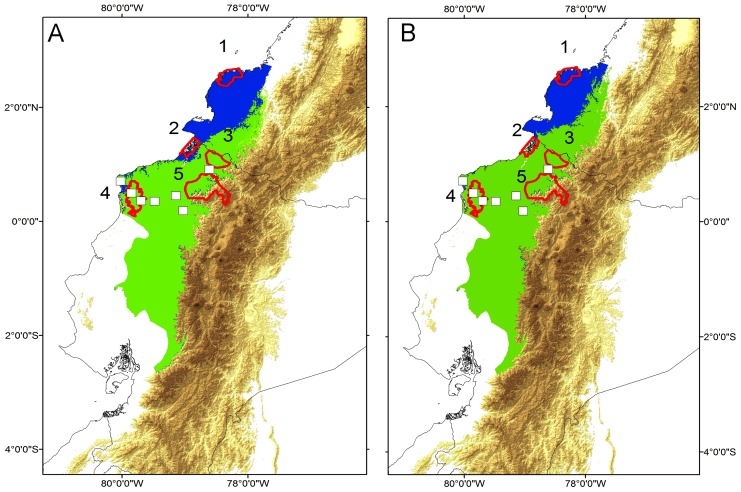
Potential distribution model of *Cochranella mache* in two future scenarios of climate change. Potential distribution model in year 2050, under the GCM’s Canadian model (A) CCCMA-A2 (“pessimistic”) and (B) CCCMA-B2 (“optimistic”) climatic scenarios. Note a reduction (∼20%, in green) of the best-predicted potential distribution model (T20 threshold), especially located in the northwestern part of the model. In both scenarios, suitability areas for *Cochranella mache* tend to loss in Sanquianga Natural National Park (1) and Manglares-Mataje Ecological Reserve (2).

Logistic threshold decision was applied on a models with a Minimum training presence (MTP) and Fixed cumulative value of 20% (T20; see Materials and methods for details). Ordered numbers correspond to the identity label in Figures 6–7.

### Impacts of deforestation on the distribution model

Intensive deforestation reduced by ∼70% the predicted potential geographic range of *Cochranella mache* (Table 3). Deforestation impacting current predicted range is most intensive across the western Andean foothills (along the roads that connect the capitals and main towns of the coastal Provinces in Ecuador and southern Colombia); on the lowland forest in Coastal region (Esmeraldas-Manabí area) and southern mountain slopes (Los Ríos, Santo Domingo, Manabí, Cotopaxi, provinces) of Central Andes (Fig. 6B). A critical reduction of 95% of the distribution model of *C. mache* is identified when combine data of deforestation and remnant model included into the limits of Protected Areas (Table 3); in contrast, about 85% of forested areas within the model is located out from the limits of Protected Areas (Fig. 6C).

### Future scenarios under climate change

Potential geographic ranges projected under two different climate change scenarios (CCCMA-A2 and CCCMA-B2 climatic models, which not consider current deforestation) for *Cochranella mache* from western Ecuador are presented on Figure 7. Reductions of the predicted geographic range caused by current deforestation and preserved in Protected Areas generated by both climate change scenarios are detailed in Table 3. Suitability areas for *Cochranella mache* are predicted to be reduced in extension, especially along the northern part of the model, mainly in lowlands of southwestern Colombia (Fig. 7). Likely to occur with the potential distribution model, the largest impact will be due by deforestation, since climate change alone will represent a net reduction of 13–21% of its future predicted geographic range (Table 3).

**Figure 8:**
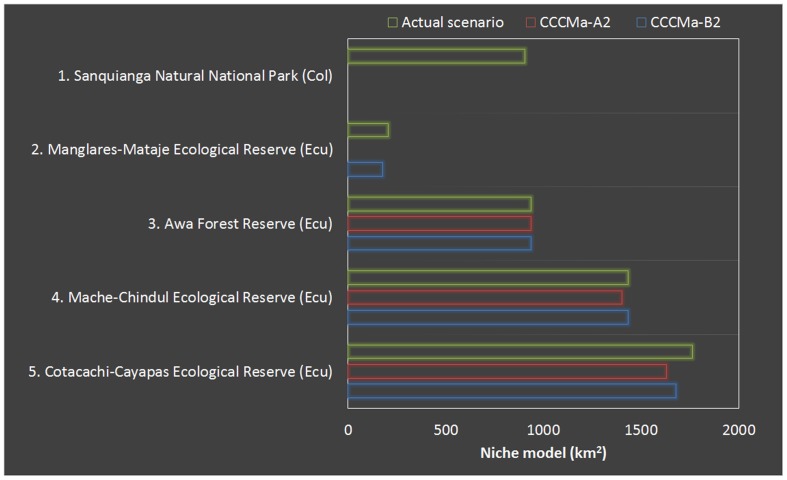
The role of protected areas in two future scenarios of climate change. In scenarios of climate change, the amount of reduction of the predicted geographic range is critical for *Cochranella mache* in Sanquianga Natural National Park (1) and Manglares-Mataje Ecological Reserve (2), whereas in the other three reserves, the reduction of the predicted potential geographic range results minimum.

The amount of reduction of the predicted geographic ranges related with Protected Areas is critical in Sanquianga Natural National Park (Colombia), either in “Pessimistic” or “Optimistic” climate change scenarios (Fig. 8). However, among Ecuadorian’ Protected Areas, a total reduction of habitat suitability is only observed in Manglares-Mataje Ecological Reserve when is evaluated within a “Pessimistic” scenario, whereas in the other three reserves the reduction of predicted potential geographic range results minimum (Fig. 8).

## Discussion

In the present study we monitored a population in northwestern Ecuador to assess abundance patterns and natural history aspects of the endangered frog, *C. mache.* We were able to find additional specimens in museum collections from two new localities and present a proposal of potential distribution model based on relevant environmental data, even in two future scenarios of climate change. This approach was useful to evaluate the effectiveness of protected areas in western Ecuador and adjacent southern Colombia, and it has the potential to be used as guide to evaluate other uncommon endangered species in the Neotropics.

### Natural history

Monitoring a *C. mache* population in Bilsa Biological Station (BBS) revealed that the species breeds at night after loud rains, during rainy season (January to June). When breeding season begins, males frequently call from midstory and canopy vertical forest stratums, or perching on leaves from surrounding vegetation up to 4 meters above ground. This species is restricted to small streams and rocky rivulets at primary and old secondary forests, with close canopy cover [16]. Cisneros-Heredia *et al.* [39] reported the first known gravid female in Canandé Biological Reserve, Esmeraldas Province, Ecuador.

Herein we described for first time the mating call for *C. mache*, being structurally similar to some distantly related species of glassfrogs (*e.g. Hyalinobatrachium fleischmanni*), mainly by the pulsed shape of waveform in the oscillogram and the range of dominant frequencies (http://zoologia.puce.edu.ec/Vertebrados/anfibios/Cantos.aspx?Id=3188). Among specific localities (small rivulets) where *C. mache* has been found, *H. fleischmanni* was not present. This could be because the latter species seems to prefer broader and torrential rivers in western Ecuador, rather than small rivers and creeks [55]. Furthermore, the reports of an amplectant pair and a gravid female (April 2009), and the abundance pattern found in Bilsa support the hypothesis that this species is a breeder throughout the rainy season, similar to other syntopic species which seems to have a different matting call structure and behavior (*e.g. Sachatamia albomaculata, Hyalinobatrachium valerioi,* [55]–[57]). No clutched eggs were seen through surveys but, it is possible that females of *C. mache* deposit their eggs on the upper sides of leaves along streams as other related species do [58].

Predation is often invoked as a population-regulating mechanism and as a factor that contributes to shape community structure [59]–[61]. The observations in the field are noteworthy because little is known about predation on centrolenids [62]. Here we describe for the first time the predation behavior on *C. mache* by a snake, *Letodeira septentrionalis*, and a Cnetid spider, *Cnetus* sp. As observed in other tropical frogs [63], snakes from genus *Leptodeira* seem to be an important predator in communities throughout the breeding season in lowland tropical forest of northwestern Ecuador [64]. Predation by spiders seems to affect early and late life history stages in *C. mache*, whereas predation on arboreal egg masses has been reported by grapsid crab (*Sesarma roberti* Edwards), phalangids (*Prionostemma frontale* Banks), and gryllids (*Paroecanthus tibialis* Saussure and Pictet) in other tropical glassfrogs [62]. The *modus operandi* by *Ctenid* spider in *C. mache* was likely similar to that described for the spider *Cupiennus* sp. (Cnetidae) on *Espadarana prosoblepon* and *Hyalionobatrachium fleischmanni*, being one of the most important predators, on adult stages, of some glassfrogs in Costa Rica [62]. More data are needed, but spiders and snakes may prove to be one of the most important predators on small terrestrial anurans and their developmental stages in riverine habitats.

High richness of glassfrogs occurs along the lowlands of extreme northwestern Ecuador, with fewer species in areas closer to the Cordillera Occidental. At least, five additional glassfrogs have been reported as sympatric with *C. mache* in rivulets in Bilsa Biological Station [34]: *Espadarana prosoblepon* (Boettger), *Sachatamia albomaculata* (Taylor), *Teratohyla pulverata* (Peters), *Hyalinobatrachium fleischmanni* Boettger, and *H. valerioi* Dunn.

### Species distribution model for *Cochranella mache*

Working on unexplored areas produces frequently new species descriptions [65]–[69], expansions of known species’ distribution ranges [70]–[73] and records of unidentified specimens on biological inventories [69], [74]. Thus, the application of technology for modeling ecological niches and predicting geographic distributions is extremely useful in defining core areas of species diversity, foci of undiscovered species and developing conservation strategies [24], [75]–[77]. In this context, modeling with small number of occurrence records needs a valuation in the fitting of the obtained model, to do a correct interpretation, identify regions with similar environmental conditions and to predict limits in the species’ distribution range [24], [78]. Herein, an exploratory model based on small sample size predicts a suitability area located from lowland forest in southwestern Colombia, through northwestern Ecuador and continued to Foothill montane forest in western Andes. An apparent over-predicted area is located in the south of the environmental suitability model, along the and manglar in Jama-Zapotillo Biogeographic Province. Since records of *C. mache* are mainly related to tropical forests in Western Ecuadorian Biogeographic Province, these predicted areas could reflects potential habitats not colonized due by biogeographic barriers [23] or areas that may conserve the niche from extinct populations [24], [27]. To prove these hypotheses, field explorations must be planning in order to assess potential new records for *C. mache* in central-western Ecuador.

As result of niche modeling, the less conservative model (T20) predicts a best potential species distribution area, which is restricted for the species (∼65,147 Km^2^) along the seasonal evergreen forests of the West Ecuadorian region, in the Cordillera Mache-Chindul, eastern Piedmont Andean forest and adjacent coastal Colombia. In the absence of local herpetological surveys, the latter area is proposed for future field surveys, as well as the Piedmont forest located in western Andes, along the limit frontier between Ecuador and Colombia.

Predicted suitability areas from small numbers of occurrence records have great results, especially in targeting field surveys to accelerate the discovery of unknown populations and species [24], [42], [79]. For example, the study of models generated for relative close phylogenetic species (*e.g. C. resplendens, C. litoralis*), which also are threatened and scarce in collections, could give us a better idea about the biogeography and the processes involved in the speciation patterns of glassfrogs in northern Andes. Moreover, the addition of new records from future field surveys has the potential to impact model predictions and clarify our understanding on the distribution of uncommon species [79], [80]. The model match with a relatively small biogeographic region, the Western Ecuador Province [32], different from the Choco and Tumbesian regions. However, further research is required to investigate population declines and targeting surveys, in order to accelerate the discovery of unknown populations on unexplored areas with high probability of relative suitability [24], [79].

As noted before, *C. mache* is a canopy dweller restricted to small streams and rivulets at primary and old secondary forest, considered as a sensitive species to habitat disturbances. In this context, a critical reduction of the potential distribution model (∼92%) is predicted after we applied a filter for Protected Areas in Ecuador and Colombia, being the Mache-Chindul and Cotacachi-Cayapas Ecological Reserves the most important for *C. mache* in the best-fitted model. Strong anthropogenic pressures have been reported for both Reserves, mainly related with an expansion of the agricultural frontier, grass for cattle, wood extraction, illegal mining and possession of land [81], [82]. Furthermore, a critical aspect found in management plans for these reserves reveals a none programmatic strategy to deal with threatened amphibians, in spite that the inventories reported that at least one third of the frogs are considered as threatened or endemic [34], [81], [82].

The suitability model predicted for *C. mache* is severely reduced when we applied the effect of habitat loss, even inside of Protected Areas (∼95% of reduction, Table 3). When the model is evaluated in two futures scenarios of climate change, it is clear a reduction in the northern part, encompassed with coastal lowland forest in Colombia and the limits along the northern frontier with Ecuador. In scenarios of climate change, the amount of reduction of the predicted geographic ranges related with Protected Areas in Sanquianga Natural National Park (Colombia) and Manglares-Mataje Ecological Reserve is critical, whereas in the other three reserves the reduction of predicted potential geographic range result minimum (Fig. 8). The fact that three reserves (Awa Forest Reserve, Mache-Chindul Ecological Reserve, Cotacachi-Cayapas Ecological Reserve) presented a minimum reduction of suitability areas on future scenarios of climate change (Fig. 8), may recommend focal sites to invest efforts in develop conservation strategies in these National Protected Areas in western Ecuador. Nevertheless, it is necessary to highlight that a synergy based on habitat loss and climate change may have significant impacts on habitat suitability for this species, even more than both factors evaluated by separated along the distributional range and Protected Areas (Table 3). In this context, our results supports other studies in which these threats, even with synergetic effects by pathogens and climate change, are potential causes for critical declines in amphibian species with restricted distributional ranges [13], [16].

Deforestation and extension of agricultural frontier are both the most important factors in habitat and biodiversity loss in Neotropics [83]–[86]. For example, as 70% of natural forests across western Ecuador have been felled [87]–[90], probably inducing changes in local climate patterns of nearby well-preserved areas and thus affected amphibians [13], [17], but with a higher impact degree due to the restricted geographic ranges of some centrolenid species [91], as probably occurs with *C. mache*.

### Conservation status of *Cochranella mache*

This frog was globally classified as “Endangered” by Guayasamin [92] under criteria B1ab(iii) because “the extent of occurrence is likely to be less than 5000 km^2^, essentially known from one location, and suspects that its habitat is undergoing continuing declines in extent, although the known range of this species is encompassed by protected areas”. Prior to this research, we only had reports from seven specimens of *C. mache* related to five localities assessed through ∼286 person/hours in samplings along northwestern Ecuador. These data was used to promote the re-categorization to Critically Endangered of the species [16]. However, Ron *et al.* [17] suggested this species should be nationally listed as Data Deficient.

New data provided in this research reveals that *C. mache* could have a wider and more abundant distribution than it was previously thought. Now we have confirmed reports of at least seven localities in northwestern of Ecuador. According to the species distribution model and historical data of distribution, this species is restricted to three vegetation formations: Foothill evergreen forests of coastal Mountains, Lowland evergreen forest and Piedmont evergreen forests of western Ecuador, being probable its presence in adjacent Colombia, in an altitudinal range up to 1000 m asl. In Ecuador, these vegetation types had a historical extension of ca. ∼46,508 km^2^; today ca. ∼75% has been severely affected by anthropogenic activities with less than 25% remaining [90], [93]–[95]. Furthermore, about 85% of the remnant distribution model for *C. mache* correspond to natural forest located out from Protected Areas.

We have at least two possible scenarios to evaluate the conservation status based on the distribution of the species: (1) conservation status assuming that the presence extension range is accurate, and (2) conservation status assuming that the distribution model is accurate. For the former scenario, the presence extension range for this species is near than 6,940 km^2^, where are extensively fragmented and continued declines of its extent and habitat quality. Since the species was described very recently [40], it is probable that the distribution of *C. mache* is likely larger than a polygon of the presence extension range, but a less number of potential undiscovered localities and subpopulations in an effective occupancy area is probable among surrounding localities, mainly by habitat loss. In a second scenario, the potential distribution model reach ∼65.147 km^2^, but a reduction in about 70% of the area is inferred only by habitat loss, being not more than 5% of the potential distribution model conserved into the SNAP (Table 3). As noted before, in both scenarios a critical reduction in the area of occurrence and ecological suitability is estimated for this species (Figs. 6–8).

According with reports of a half of centrolenid species which are declining and threatened with extinction [16], [17], [96], [97], we suspect that a reduction ≥ 50% of the population size is possible in the next decades. Moreover, based on the predicted model of distribution, the current extension or remaining ecological suitable habitats in Protected Areas range is less than 5000 km^2^, being progressively reduced by extreme fluctuation in forest coverture in nearby areas of Ecological reserves. Besides, the potential effect of climate change might represent a synergetic risk, especially in the northern part of the model, where few Protected Areas are located (Figs.7–8).

Considering these arguments, we recommend that *C. mache* must be re-categorized as “Critically Endangered” species in national and global status, according with criteria and sub-criteria A4, B1ab(i,ii,iii,iv),E [51], based in a probable extinction ≥ 50% of local populations in the next generations by synergetic effects of habitat loss and climate change.
