# Correction: Novel Data on the Ecology of *Cochranella mache* (Anura: Centrolenidae) and the Importance of Protected Areas for This Critically Endangered Glassfrog in the Neotropics

**DOI:** 10.1371/annotation/3afad533-ccf9-4d29-83ea-e4eeae57203c

**Published:** 2013-12-20

**Authors:** H. Mauricio Ortega-Andrade, Octavio Rojas-Soto, Christian Paucar

Figures 4 and 5 were incorrectly switched. The image currently appearing as Figure 4 belongs with the title and legend for Figure 5, and the image currently appearing as Figure 5 belongs with the title and legend for Figure 4. The titles and legends are in correct order.

